# In Patients Hospitalized for Community-Acquired Pneumonia, SARS-CoV-2 Is Associated with Worse Clinical Outcomes When Compared to Influenza

**DOI:** 10.3390/pathogens12040571

**Published:** 2023-04-07

**Authors:** Jeffrey Spindel, Stephen Furmanek, Thomas Chandler, Julio A. Ramirez, Rodrigo Cavallazzi

**Affiliations:** 1 Gill Heart and Vascular Institute, University of Kentucky, Lexington, KY 40536, USA; 2 Norton Infectious Diseases Institute, Norton Healthcare, Louisville, KY 40202, USA; 3 Division of Pulmonary, Critical Care Medicine, and Sleep Disorders, University of Louisville, Louisville, KY 40202, USA

**Keywords:** COVID-19, influenza, pandemic, invasive mechanical ventilation, nursing home

## Abstract

SARS-CoV-2 and influenza are primary causes of viral community-acquired pneumonia (CAP). Both pathogens have exhibited high transmissibility and are recognized causes of pandemics. Controversy still exists regarding the clinical outcomes between patients hospitalized with CAP due to these viruses. This secondary analysis identified patients with either influenza or SARS-CoV-2 infections from three cohorts of patients hospitalized for CAP. Clinical outcomes between patients with CAP due to influenza or due to SARS-CoV-2 were evaluated. Primary outcomes included length of stay and in-hospital mortality. To account for population differences between cohorts, each case of influenza CAP was matched to two controls with SARS-CoV-2 CAP. Matching criteria included sex, age, and nursing home residency. Stratified cox-proportional hazards regression or conditional logistic regression were used where appropriate. A total of 259 patients with influenza CAP were matched to two controls with SARS-CoV-2 CAP, totaling to 518 controls. Patients with SARS-CoV-2 CAP were 2.23 times more likely to remain hospitalized at any point in time (95% confidence interval: 1.77–2.80), and had 3.84 times higher odds of dying in-hospital (95% confidence interval: 1.91–7.76) when compared to patients with influenza CAP. After matching and adjusting for confounding variables, patients admitted with SARS-CoV-2 CAP had consistently worse outcomes in comparison to their influenza CAP counterparts. This information can help clinicians decide on the level of care needed for patients with confirmed infections due to these pathogens. Additionally, estimates of disease burden can inform individuals at-risk for poor clinical outcomes, and further highlight the importance of effective preventative strategies.

## 1. Introduction

Influenza is a common cause of community-acquired pneumonia (CAP) and a major public health burden. Influenza pandemics have been documented since the middle ages, with the worst influenza pandemic occurring in 1918 which resulted in 50 million deaths worldwide [1]. SARS-CoV-2 emerged in 2019 as the virus responsible for the COVID-19 pandemic and a major cause of death due to CAP [2]. In the United States (US), deaths attributed to SARS-CoV-2 far exceed deaths during the influenza pandemic of 1918 [1,2]. As rates of prior SARS-CoV-2 infection and vaccination administration increase within the general population, rates of hospitalization and mortality from SARS-CoV-2 CAP in the US have decreased [3]. However, new variants of SARS-CoV-2 continue to emerge, and breakthrough infections are reported in previously infected and/or vaccinated individuals [4,5,6,7].

When comparing the potential origins of pandemic strains of influenza and SARS-CoV-2, both pathogens exhibit transmissibility between humans and animals [8]. However, pandemic strains of influenza emerged solely from animal hosts via antigenic shift prior to human-to-human transmission [9]. In contrast to influenza, SARS-CoV-2 has shown the ability to produce significant mutations following human-to-human transmissions, often resulting in increased transmissibility [10]. Therefore, SARS-CoV-2 may likely become a recurrent, seasonal infection in a similar fashion to influenza, adding to the concern for future outbreaks and subsequent pandemics [11].

Although the landscape of the SARS-CoV-2 pandemic continues to evolve, higher mortality due to SARS-CoV-2 CAP has been extensively reported in nursing home residents, elderly persons, and males. Previously reported data estimated that nursing home residents accounted for 5% of SARS-CoV-2 infections, but nearly 40% of the deaths due to SARS-CoV-2 [12,13]. When compared to earlier estimates, current rates of hospitalization and mortality have decreased due to vaccination efforts and differences in viral variants [14,15,16,17]. However, seasonal variants of influenza can still exhibit lethality despite vaccination status [18], suggesting the potential for a similar scenario as new SARS-CoV-2 variants emerge.

Consequently, a better understanding of the burden of influenza and SARS-CoV-2 CAP are important for both clinicians and public health officials in the US. Therefore, a comparison of the outcomes of patients with SARS-CoV-2 and influenza CAP that accounts for underlying population differences is needed. The primary aim of this study is to define differences in hospital length of stay and in-hospital mortality between patients hospitalized for influenza and SARS-CoV-2 CAPS. Secondary aims of this study include comparing clinical presentation, admission or transfer to intensive care unit (ICU), need for mechanical ventilation, and cardiovascular events.

## 2. Materials and Methods

This was a secondary, matched analysis of data from three cohort studies: The Rapid Empiric Treatment with Oseltamivir Study (RETOS), the University of Louisville Pneumonia Study (ULPS), and the Burden of COVID-19 study [19,20,21]. RETOS was a clinical trial which evaluated the use of oseltamivir as empiric treatment for patients with lower respiratory tract infections. RETOS was performed at all nine acute-care hospitals in Louisville, Kentucky from 2010 to 2013. The ULPS was a prospective cohort study that enrolled hospitalized patients with CAP between 2014 and 2016 at any of the nine adult acute-care hospitals in Louisville, KY [21]. For this matched analysis, subjects from the RETOS or ULPS and hospitalized for influenza CAP were identified. The Burden of COVID-19 study enrolled patients with SARS-CoV-2 CAP from September 2020 to March 2021 [22]. In this analysis, comparisons were made between the following groups: (1) SARS-CoV-2 CAP, and (2) influenza CAP.

To account for temporal differences, only subjects hospitalized with CAP during influenza seasons (October–March) were included. Subjects with influenza infections in RETOS and ULPS were confirmed by polymerase chain reaction (PCR) testing of a oropharyngeal or nasopharyngeal swab, or by rapid influenza antigen testing. Subjects in the Burden of COVID-19 study had a confirmed SARS-CoV-2 infection by a positive reverse transcriptase PCR test of a nasopharyngeal swab.

Subjects in RETOS had a CAP diagnosis defined by the presence of two respiratory signs or symptoms, and one sign of an acute infection at the time of admission. Respiratory signs and symptoms were new or increased cough, change in sputum production, oxygen saturation < 90% on room air or a 1 L/min increase from home oxygen requirements, new dyspnea, tachypnea (>24 breaths per minute), or new crackles, rhonchi, or wheezing on pulmonary auscultation. Signs of acute infection included fever > 38 °C, hypothermia < 35.6 °C, subjective fever, chills, myalgia, altered mental status, and one of leukocytosis, leukopenia, left shift, or lymphocytosis. All subjects had signs of pulmonary infiltrate on a chest radiograph and/or chest computed tomography (CT). All nasopharyngeal swabs were obtained at subject enrollment, with testing for 12 respiratory viruses including influenza [20].

The ULPS and the Burden of COVID-19 studies defined CAP as the presence of new pulmonary infiltrate on chest radiograph and/or CT; one of: new or worsening cough, increased sputum production, fever > 37.8 °C, hypothermia < 35.6 °C, leukocytosis > 11,000 cells/μL, left shift > 10% bands, or leukopenia; and no alternative diagnosis to account for the above criteria. Microbiological testing and workup for subjects in ULPS were performed at the discretion of the treating clinician.

Study variables abstracted from electronic medical records included demographics, comorbidities, signs and symptoms, physical examination and laboratory findings, and severity of illness at the time of admission. Severity of illness at the time of admission was defined by the presence of the following: altered mental status, ICU care, invasive mechanical ventilation (IMV), non-invasive mechanical ventilation (NIMV), vasopressors use and pleural effusion. Admitting severity was also quantified using the Pneumonia Severity Index (PSI). Originally defined by Fine et al., PSI scores are assigned from a patient’s age and medical comorbidities and stratified into risk classes. These strata can be used to estimate a patient’s risk for 30-day mortality following an initial CAP hospitalization [23,24].

The primary outcomes compared were hospitalized length of stay and in-hospital mortality. Length of stay (LOS) was determined as the length of hospitalization from admission to discharge. LOS was right truncated at 30 days for every patient in order to capture only CAP-related length of stay. Patients who died during hospitalization were given a censored at 30 days for all cohorts. In-hospital mortality was defined as all-cause mortality during hospitalization.

Secondary outcomes assessed included the following events at any point during hospitalization: ICU stay, IMV, NIMV, vasopressor use, and cardiovascular events. Cardiovascular events included acute myocardial infarction, pulmonary edema, new arrhythmia, acute worsening of chronic arrhythmia, cerebrovascular accident, and pulmonary embolism.

Subjects hospitalized due to influenza CAP in the RETOS and ULPS databases were matched to two subjects hospitalized due to SARS-CoV-2 CAP from the Burden of COVID-19 study to balance on baseline covariates. Matching criteria included sex, age (in 5-year increments), and nursing home status. A greedy, exact-matching algorithm was employed to prevent the subsequent matching of previously matched. Descriptive statistics were reported as median and interquartile range for continuous values. Categorical values were reported as frequency and percentage. Due to violations of the independent observations assumption in the matched dataset, statistical testing on baseline characteristics was not performed. Standardized mean differences (SMDs) were reported after matching to assess balance of baseline characteristics. Conditional logistic regressions and stratified proportional hazards regressions were used to account for matching when assessing primary and secondary outcomes. Characteristics that remained imbalanced after matching were adjusted for in the regression models, and included PSI, BMI, and COPD. Conditional odds ratios (cOR) and stratified hazard ratios (sHR) were reported for primary and secondary outcomes with 95% confidence intervals. Statistical analysis was performed using R version 3.4.2 [25]. *p*-values of less than 0.05 were considered statistically significant.

## 3. Results

**Figure 1:**
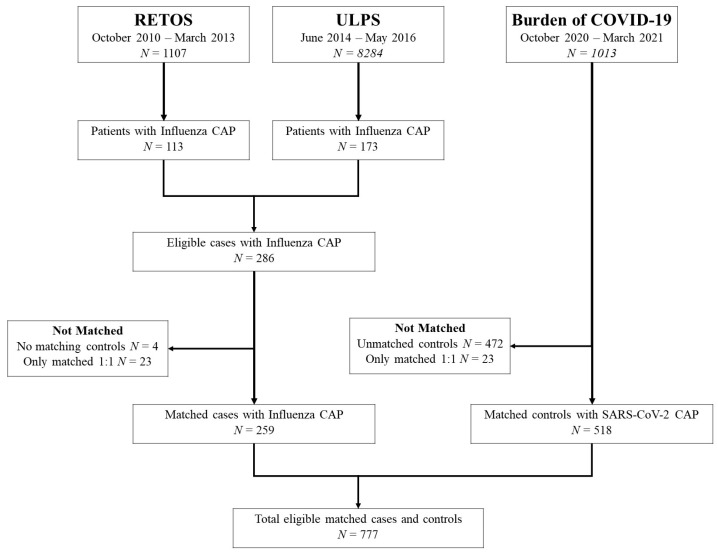
Patient Selection Flowchart.

The matched dataset consisted of 777 cases and controls. Cases included 259 patients hospitalized with influenza CAP identified from the RETOS and ULPS studies. Each case of influenza CAP was matched to two controls from the Burden of COVID-19 study, totaling 518 subjects with SARS-CoV-2 CAP. Figure 1 outlines patient selection from the three studies included in this analysis.

### 3.1. Medical History

Patient characteristics are depicted in Table 1. The median age was 64 years for influenza and SARS-CoV-2 CAP. Nursing home residents made up 6% of the population in both groups. Fewer patients hospitalized with influenza CAP were obese (39% vs. 59%, SMD = 0.402). In contrast, a higher prevalence of renal disease, heart failure, chronic obstructive pulmonary disease (COPD), liver disease, and current smoking status was observed among patients with influenza CAP. There were no differences in rates of HIV, active cancer status, stroke, or diabetes between groups.

### 3.2. Signs and Symptoms

Reported symptoms and physical exam findings are depicted in Table 2. Dyspnea was frequently reported in patients with influenza CAP and SARS-CoV-2 CAP (82% vs. 76%, SMD = 0.138). Patients with influenza CAP reported sputum production (50% vs. 17%, SMD = 0.730), crackles, wheezes, or rhonchi (43% vs. 27%, SMD = 0.339), new or worsening cough (85% vs. 69%, SMD = 0.370) than patients presenting with SARS-CoV-2 CAP.

### 3.3. Laboratory Findings

Laboratory findings are shown in Table 3. Elevated values for blood urea nitrogen were observed among patients with SARS-CoV-2 CAP (17 vs. 19; SMD = 0.226). While hematocrit and platelet count were similar, white blood cell count (WBC) was lower in the SARS-CoV-2 cohort (9.8 vs. 7.0, SMD = 0.461). Median highest recorded serum C-reactive protein was elevated at 34.0 mg/L [IQR 12.6, 103.5] and IL-6, D-dimer, and ferritin were elevated in the SARS-CoV-2 cohort. Inflammatory markers are shown in Supplemental Table S1. Since measuring inflammatory markers in the setting of influenza pneumonia is not standard of practice, comparable data is not available for our influenza cohort. Arterial oxygen saturation to inhaled oxygen percentage (PaO_2_/FiO_2_) ratio was significantly lower in the SARS-CoV-2 cohort, indicating a higher degree of alveolar-arterial oxygen gradient or ventilation–perfusion mismatch.

### 3.4. Severity of Illness on Admission

Severity of illness on admission is presented in Table 4. Patients with influenza CAP presented with pleural effusion (25% vs. 16%; SMD = 0.212) more frequently. Patients with SARS-CoV-2 CAP were admitted to the ICU more frequently (18% vs. 11%, SMD = 0.202). Vasopressors use on the first day of admission was also more frequent among patients with SARS-CoV-2 CAP (6% vs. 3%, SMD = 0.154). Differences in altered mental status, need for IMV, need for NIMV, and PSI risk class were not observed at the time of admission.

### 3.5. Primary Outcomes

The median length of stay for influenza CAP was shorter when compared to SARS-CoV-2 CAP (4 days vs. 8 days, SMD = 0.669). After matching and adjusting for PSI, BMI, and COPD, patients with influenza CAP were 2.23 times more likely to be discharged from the hospital at any timepoint, when compared to patients with SARS-CoV-2 CAP (sHR: 2.23, 95% CI: 1.77–2.80, *p*-value < 0.001).

In-hospital mortality was lower among patients with influenza CAP when compared to patients with SARS-CoV-2 CAP (6% vs. 16%, SMD = 0.338). After matching and adjusting, the conditional odds of in-hospital mortality was 3.84 times higher among patients with SARS-CoV-2 CAP (cOR: 3.84; 95% CI: 1.91–7.76 *p*-value < 0.001) when compared to the influenza CAP counterparts.

### 3.6. Secondary Outcomes

**Figure 2:**
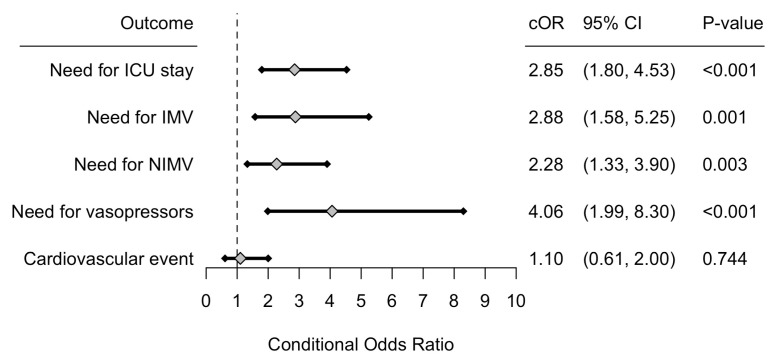
Forrest plot of the conditional odds ratios (cOR), corresponding 95% confidence interval (CI) and *p*-value for all secondary outcomes. Interpretation should be made for patients with SARS-CoV-2 CAP, and use influenza CAP as the reference level. Abbreviations: CAP: Community-acquired pneumonia, ICU: Intensive care unit, IMV: invasive mechanical ventilation, NIMV: Non-invasive mechanical ventilation.

Secondary outcomes are presented in Figure 2. ICU care at any point (direct ICU admission or transfer) during hospitalization was 2.85 (95% CI: 1.80–4.53, *p*-value < 0.001) times higher among patients admitted with SARS-CoV-2 CAP. The odds that a patient required IMV, NIMV, and vasopressors use during hospitalization was 2.88 (95% CI: 1.58–5.25, *p*-value = 0.001) times higher, 2.28 (95% CI: 1.33–3.90, *p*-value = 0.003) times higher, and 4.06 (95% CI: 1.99–8.30, *p*-value < 0.001) times higher among patients with SARS-CoV-2 CAP, respectively. As a group, cardiovascular outcomes were not significantly different among groups. Specific cardiovascular complications are presented in Table 5.

While ICU level of care at any point during hospitalization was significantly more frequent in the SARS-CoV-2 group, illness severity at presentation was comparable, detailing that these patients had significantly higher rates of clinical deterioration after hospital admission.

## 4. Discussion

Our findings indicate that mortality among patients hospitalized with SARS-CoV-2 CAP is almost four times higher than mortality among patients hospitalized with influenza CAP. Additionally, patients with SARS-CoV-2 CAP had nearly twice the median length of stay when compared to patients with influenza CAP. At any given point in time, patients with SARS-CoV-2 CAP were almost 2.5 times more likely to remain in the hospital when compared to influenza CAP patients.

Patients with influenza CAP in our cohort had more comorbidities such as COPD, liver disease, renal disease, heart failure, and were more frequently current smokers. In addition to increased comorbidity, patients with influenza CAP presented with signs and symptoms more frequently. In contrast, patients admitted with SARS-CoV-2 CAP were less comorbid, but more frequently obese than those admitted with influenza CAP. Obesity is a known risk factor for SARS-CoV-2 infection, requiring hospitalization, and developing a more severe illness that requires mechanical ventilation. These poor outcomes may result from an abnormal immune response and metabolic dysfunction seen in obese individuals [26]. Our data support this finding.

We hypothesize that patients with SARS-CoV-2 have increased in-hospital mortality due to heightened or dysfunctional inflammatory state and ventilation–perfusion mismatch. The heightened or dysfunctional inflammatory state has been well detailed in the literature as the cytokine storm. Higher serum cytokine levels are associated with shorter survival as well as multiorgan damage [27,28]. However, it is not known if immune hyperactivity is due purely to immune dysregulation or as a response to ongoing viral replication. Reduced WBC have been reported in the setting of SARS-CoV-2 infection with lower counts correlating with worse disease severity and outcomes [29]. In our sample, WBC were lower in the SARS-CoV-2 cohort compared to the influenza cohort. Lower regulatory lymphocytes may contribute to immune dysregulation and have been shown to inversely correlate with cytokine levels during cytokine storm, suggesting that immune hyperactivity and dysregulation occur concomitantly [30]. While we do not have inflammatory markers from our cohort with influenza CAP, serum levels suggest that cytokine storm had a role in the higher morbidity seen in our SARS-CoV-2 cohort.

Ventilation–perfusion mismatch is multifactorial. Besides the ventilatory impairment inherent with pneumonia and adult respiratory distress syndrome, SARS-CoV-2 has also been found to cause perfusion mismatch. Autopsy of patients who died from SARS-CoV-2 demonstrated severe endothelial injury associated with intracellular virus. Furthermore, histologic analysis revealed widespread thrombosis with microangiopathy, specifically alveolar capillary microthrombi. This phenomenon, termed endothelialitis, was 9 times more prevalent in the tissue of patients who died from SARS-CoV-2 than patients who died from influenza [31]. In our cohort, PaO_2_/FiO_2_ ratio was significantly worse in the SARS-CoV-2 cohort, supporting a higher degree of ventilation–perfusion mismatch. Interestingly, patients with SARS-CoV-2 CAP were less symptomatic and similarly ill at the time of admission, but more frequently deteriorated, subsequently required ICU level care, and died in-hospital. This suggests that endothelialitis and the higher degree of ventilation–perfusion mismatch occur later in the disease course, and supports an early intervention with the available therapies.

Our data refute the impression that outcomes related to SARS-CoV-2 worsen due to an older, frailer, or more comorbid population. This analysis was performed on a dataset that matched nursing home residency, sex, and age as a method to hold any confounding effects to constant. Additionally, the estimates produced from the multivariate analysis also accounted for pneumonia severity, obesity, and COPD. Our findings lend strong support to the notion that hospitalized patients with SARS-CoV-2 CAP have worse outcomes compared to those hospitalized with influenza CAP in the general population.

Comparative analyses of the clinical presentation and outcomes of CAP caused by SARS-CoV-2 or influenza have reported conflicting results. In a French study of 124 patients, Zayet et al. reported no differences in rates of admission to ICU, hospital LOS, comorbidities, and outcomes [32]. In a U.S. study of 65 critically ill patients, Cobb et al. reported no difference in rates of invasive mechanical ventilation between patients with SARS-CoV-2 or influenza CAP, though patients with SARS-CoV-2 had longer duration of mechanical ventilation. Mortality rates were also higher among patients with SARS-CoV-2 CAP (40% vs. 18.9%). In concordance with our findings, patients with influenza CAP had higher rates of COPD and tobacco use, whereas patients with SARS-CoV-2 had higher BMI [33]. Piroth et al. conducted a population-based study in France which compared all patients admitted with SARS-CoV-2 CAP from March-April 2020 to patients from a nationwide database during the 2018-2019 influenza season. The authors determined that patients admitted with SARS-CoV-2 CAP had higher rates of hypertension, diabetes, dyslipidemia, and BMI ≥ 25 kg/m^2^, whereas patients admitted with influenza CAP had higher rates of heart failure, chronic respiratory disease, cirrhosis, anemia, and immunodeficiencies. Rates of invasive mechanical ventilation, acute kidney failure, ICU admission, ICU LOS were also higher among patients SARS-CoV-2 CAP. The authors also reported a higher mortality rate of 16.9% among SARS-CoV-2 CAP, in comparison to 5.8% among influenza CAP [34]. Despite the large number of patients, the study was conducted prior to available vaccinations, in adults and children, and in a population with different rates of comorbidities and community living than the American population.

Our study is still subject to several limitations. First, data was collected from different years in the three cohorts of patients included in the analysis. However, only patients hospitalized for SARS-CoV-2 pneumonia during the influenza season were analyzed to make the cohorts more comparable. Next, this study represents patients hospitalized in one city, but it includes patients from multiple hospitals. The inclusion of only one city may limit external validity. However, we have previously demonstrated that the demographics of Louisville are similar to the overall US [21]. Additionally, one drawback of exact matching is that not all patients in the study cohorts were able to be matched. However, the use of a conditional logistic regression allows for a balanced analysis by accounting for both the matching criteria and additional confounding variables. Simply analyzing all patients in an unmatched cohort may produce unreliable estimates.

Furthermore, we could not account for vaccination rates in the influenza or SARS-CoV-2 cohorts. Prior vaccination has shown to reduce the severity of illness in both influenza and SARS-CoV-2 infected individuals [35,36]. Patients in the SARS-CoV-2 CAP cohort were hospitalized during a time of the COVID-19 pandemic when current vaccinations were not widely available. It is possible that the availability of seasonal influenza vaccinations at the time of the RETOS and HAPPI studies may confound our results. However, the high mortality rate among unvaccinated patients with SARS-CoV-2 CAP in our study highlights the need for effective vaccination strategies.

Lastly, viral variants have differing rates of transmissibility, antibody-mediated response, and different spectrums of disease severity [37,38]. Because of this, our outcomes may not be applicable to all disease strains. However, our data represents multiple years of admissions due to influenza CAP and therefore encompasses many strains of the virus. Our data regarding SARS-CoV-2 CAP also represents a period with multiple active viral strains, which should increase the future applicability of our study. Further research is necessary to strengthen understanding of outcomes as the pandemic progresses.

## 5. Conclusions

Patients hospitalized with SARS-CoV-2 CAP were less comorbid and experienced fewer signs and symptoms than patients with influenza CAP; however, the rate of poor clinical outcomes among patients with SARS-CoV-2 CAP remained alarmingly high. Hospital facilities and providers treating high volumes of SARS-CoV-2 CAP should prepare for prolonged hospital stays, additional utilization of resources such as mechanical ventilation, and anticipate having to communicate poor prognosis. Public health professionals should continue to communicate updated estimates of disease burden associated with SARS-CoV-2 to the general public, and advocate for at-risk populations when allocating preventative interventions such as immunization.

## Figures and Tables

**Table 1:** Baseline characteristics between patients with Influenza CAP and SARS-CoV-2 CAP.

Variable	Influenza CAP (*n* = 259)	SARS-CoV-2 CAP (*n* = 518)	SMD
Age, median [IQR]	64 [54, 75]	64 [54, 75]	0.003
Male sex, n (%)	126 (49)	252 (49)	<0.001
Nursing home resident, n (%)	16 (6)	32 (6)	<0.001
COPD, n (%)	127 (49.0)	95 (18.3)	0.687
Current smoker, n (%)	81 (31.5)	38 (7.4)	0.641
Obese (BMI > 30), n (%)	101 (39.0)	304 (58.7)	0.402
Heart failure, n (%)	62 (23.9)	68 (13.1)	0.281
Liver disease (non-cirrhotic), n (%)	21 (8.1)	18 (3.5)	0.199
HIV, n (%)	6 (2.3)	2 (0.4)	0.168
Renal disease, n (%)	66 (25.5)	104 (20.1)	0.129
Diabetes, n (%)	100 (38.6)	219 (42.3)	0.075
Cerebrovascular disease, n (%)	29 (11.2)	52 (10.0)	0.038
Neoplastic disease (active or within past year), n (%)	26 (10.0)	55 (10.6)	0.019

Abbreviations: IQR: Interquartile range, CAP: Community-acquired pneumonia, SMD: Standardized mean difference, COPD: Chronic obstructive pulmonary disease, BMI: Body mass index, HIV: Human immunodeficiency disease, COPD: Chronic obstructive pulmonary disease.

**Table 2:** Signs and symptoms of patients with Influenza CAP and SARS-CoV-2 CAP.

Variable	Influenza CAP (*n* = 259)	SARS-CoV-2 CAP (*n* = 518)	SMD
Sputum production, n (%)	108 (49.8)	90 (17.4)	0.730
New or worsening cough, n (%)	198 (84.6)	359 (69.3)	0.370
Presence of crackles, wheezes, or rhonchi, n (%)	92 (43.0)	140 (27.0)	0.339
Chest pain, n (%)	63 (29.6)	82 (15.8)	0.333
Dyspnea, n (%)	183 (81.7)	394 (76.1)	0.138
Fever, n (%)	145 (56.0)	258 (49.8)	0.124

Abbreviations: IQR: Interquartile range; CAP: Community-acquired pneumonia, SMD: Standardized mean difference.

**Table 3:** Clinical and laboratory values of patients with SARS-CoV-2 CAP and Influenza CAP.

Variable	Influenza CAP (*n* = 259)	SARS-CoV-2 CAP (*n* = 518)	SMD
Heart Rate (beats/min), median [IQR]	107.0 [93.0, 120.0]	98.0 [83.0, 109.2]	0.226
Blood urea nitrogen (mg/dL), median [IQR]	17.0 [12.5, 26.0]	19.0 [13.0, 31.9]	0.226
Respiratory Rate (breaths/min), median [IQR]	22.0 [20.0, 26.0]	24.0 [20.0, 29.0]	0.124
Sodium (mEq/L), median [IQR]	137.0 [134.0, 139.0]	136.0 [133.0, 139.0]	0.114
Systolic Blood Pressure (mmHg), median [IQR]	124.0 [102.5, 144.0]	122.0 [108.0, 138.0]	0.084
Diastolic Blood Pressure (mmHg), median [IQR]	61.0 [51.0, 73.5]	63.0 [53.0, 73.2]	0.074
Hematocrit (%), median [IQR]	37.8 [33.4, 41.0]	38.0 [33.8, 41.6]	0.068
WBC × 1000 per μL (median [IQR])	9.8 [6.6, 13.4]	7.0 [4.7, 10.4]	0.461
Platelets × 1000 per μL (median [IQR])	194.0 [151.5, 253.5]	204.0 [162.5, 277.0]	0.155
Temperature (degrees C), median [IQR]	100.0 [98.8, 101.6]	99.1 [98.4, 100.6]	0.055
Glucose (mg/dL), median [IQR]	141.0 [110.2, 199.5]	138.0 [114.0, 190.0]	0.011
* PaO_2_/FiO_2_ (median [IQR])	225.1 [170.2, 294.0]	162.5 [75.6, 258.9]	0.075

Abbreviations: IQR: Interquartile range, CAP: Community-acquired pneumonia, WBC: white blood cell count, PaO_2_: arterial oxygen saturation, FiO_2_: fraction of inspired oxygen. SMD: Standardized mean difference. * Data not available in 176 and 315 patients in the respective cohorts.

**Table 4:** Severity of Illness on Admission between Influenza CAP and SARS-CoV-2 CAP.

Variable	Influenza CAP (*n* = 259)	SARS-CoV-2 CAP (*n* = 518)	SMD
Pleural effusion, n (%)	64 (25)	84 (16)	0.212
Direct admission to the ICU, n (%)	29 (11)	95 (18)	0.202
Vasopressor use on admission, n (%)	7 (3)	30 (6)	0.154
Need for invasive ventilation, n (%)	12 (4.6)	32 (6.2)	0.068
Altered mental status, n (%)	30 (12)	69 (13)	0.054
PSI, median [IQR]	91.0 [66.0, 116.5]	90.5 [66.0, 115.0]	0.018
Need for non-invasive ventilation, n (%)	17 (6.6)	33 (6.4)	0.008

Abbreviations: CAP: Community-acquired pneumonia, SMD: Standardized mean difference, ICU: Intensive care unit, PSI: Pneumonia severity index, IQR: Interquartile range.

**Table 5:** In-hospital cardiovascular events between Influenza CAP and SARS-CoV-2 CAP.

Variable	Influenza CAP (*n* = 259)	SARS-CoV-2 CAP (*n* = 518)	SMD
Pulmonary embolism (%)	0 (0.0)	12 (2.3)	0.218
Acute myocardial infarction (%)	2 (0.8)	11 (2.1)	0.113
Cerebrovascular disease (%)	1 (0.4)	6 (1.2)	0.088
Pulmonary edema (%)	4 (1.5)	4 (0.8)	0.072
Acute arrhythmia (%)	15 (5.8)	24 (4.6)	0.052
Acute worsening of long-term arrhythmia (%)	4 (1.5)	8 (1.5)	<0.001
Any cardiovascular event (%)	24 (9.3)	57 (11.0)	0.058

Abbreviations: CAP: Community-acquired pneumonia, SMD: Standardized mean difference.

## Data Availability

Not applicable.

## Supplementary Materials

The following supporting information can be downloaded at: https://www.mdpi.com/article/10.3390/pathogens12040571/s1, Table S1. Highest recorded inflammatory markers.
