# Supplementary material for: In Patients Hospitalized for Community-Acquired Pneumonia, SARS-CoV-2 Is Associated with Worse Clinical Outcomes When Compared to Influenza
Source: Pathogens. 2023 Apr 7;12(4):571. doi: 10.3390/pathogens12040571 (PMC10142714; doi:10.3390/pathogens12040571)
Supplement: Supplementary file 1 [file pathogens-12-00571-s001.zip › pathogens-2252307-supplementary.pdf]

Supplemental Table S1. Highest recorded inflammatory markers.

	Influenza CAP	SARS-CoV-2 CAP	SMD
	259	518	
CRP mg/L (median [IQR])	86.7 [61.2, 170.8]	34.0 [12.6, 103.5]	0.519
Missing (n)	*253*	*119*	
D-Dimer ng/mL (median [IQR])	NA [NA, NA]	803.0 [356.0, 1677.0]	
Missing (n)	*259*	*137*	
Ferritin ng/mL (median [IQR])	NA [NA, NA]	418.0 [222.0, 774.0]	
Missing (n)	*259*	*137*	
IL-6 pg/mL (median [IQR])	NA [NA, NA]	30.4 [12.7, 82.0]	
Missing (n)	*259*	*356*	

[1] **CRP: C-reactive protein, IL-6: interleukin-6, SMD: standardized mean difference**
